# The Brazilian army and the low prevalence of sexually transmitted infections in women of the military garrison of Campinas between 2017 to 2020: a prospective, cross-sectional epidemiological study

**DOI:** 10.1590/1516-3180.2022.0557.R1.010623

**Published:** 2023-07-31

**Authors:** Fabia Lopes, Fernanda Kesselring Tso, Neila Maria de Góis Speck

**Affiliations:** I MD. Doctoral Student and Brazilian Army Medical Captain, Command of the 11th Mechanized Infantry Brigade, Universidade Federal de São Paulo (UNIFESP), São Paulo (SP), Brazil.; II MD, PhD. Assistant Physician, Department of Gynecology, Universidade Federal de São Paulo (UNIFESP), São Paulo (SP), Brazil.; III MD, MSc, PhD. Adjunct Professor, Department of Gynecology, Universidade Federal de São Paulo (UNIFESP), São Paulo (SP), Brazil.

**Keywords:** Infections, Prevalence, Sexually transmitted diseases, Brazilian army, Military, Sexually transmitted infections, Military women, Chlamydia

## Abstract

**BACKGROUND:**

Given the characteristics of military missions, intense interpersonal contact, and wide variation in casual relationships, the military has long been recognized as a high-risk population for sexually transmitted infections (STIs).

**OBJECTIVE:**

To assess the prevalence of STIs and socioepidemiological profile of women in the military garrison of Campinas.

**DESIGN AND SETTING:**

This prospective, cross-sectional epidemiological study, assisted by the Health Fund in the military garrison of Campinas, assessed the prevalence of human immunodeficiency virus (HIV), hepatitis B and C, syphilis, human papillomavirus (HPV), *chlamydia*, and *gonococcus* in military women or companions of soldiers with active or previously active sexual life.

**METHODS:**

This study included 647 women based on the non-inclusion criteria. They underwent clinical and laboratory tests for diagnosis of STIs. For statistical analysis, patients were divided into groups according to the presence or absence of STIs and into age groups.

**RESULTS:**

Most women were military dependents, and the majority were asymptomatic. The prevalence of STIs, in ascending order, was 0.3% for hepatitis B and C, 0.62% for syphilis, 0.62% for gonorrhea, 1.08% for chlamydia, and 2.63% for HPV. There were no cases of HIV infection.

**CONCLUSIONS:**

The Brazilian Army has the most women-like dependents in the military, belonging to the hierarchical circle of the squares. Early onset of sexual activity favored STIs approximately twice, and younger women had approximately seven times more chlamydia infections. In the general population studied, the prevalence of STIs was lower than expected than in the armed forces of other nations.

## INTRODUCTION

In the armed forces, the characteristics of the missions, intense interpersonal interactions, wide variations in casual relationships, and long periods of abstinence and transit favor sexually transmitted infections (STIs). Historically, STI rates among military personnel in the United States have been higher than those among civilians.^1^

Young male soldiers are likely to be the main vectors of STIs, which can increase transmission to young military women and dependents, since close coexistence can stimulate affective and sexual bonds. Irregular use of condoms can also have a significant impact. Chao et al. demonstrated that 25% of the Brazilian population had sexarche before 15 years of age, and another 35% between 15–19 years of age. In the same survey, the regular use of condoms was unsatisfactory, with only a 39% use among people aged 15–64 years.^2–4^

A large survey of women in the United States military identified a 9.2% prevalence of chlamydia infection among female recruits, with a peak of 12.2% at 17 years of age. Prevalence declined markedly with increasing age, dropping to 5% for women over 25 years of age.^5^

In Brazil, there are few studies relating military behavior to STIs or any other diseases. Since 1996, the Brazilian Department of Sexually Transmitted Infections, STI-AIDS and Viral Hepatitis, has conducted probability sampling surveys to determine the prevalence of human immunodeficiency virus (HIV), hepatitis B and C, and syphilis by assessing sexual and risk behaviors among recruits aged 17–22 years old from the Brazilian Army using questionnaires.^6–8^

Currently, one of the major concerns in the Brazilian Army is the sexual health of women who begin their military career very early (between 17 and 20 years of age in the military line).^9^

According to the Pan American Health Organization, half of the new HIV infections arise in children under 24 years of age, with the majority being sexually transmitted.^10,11^

According to a recent study in the United States, STIs are on the rise in the United States military, and women are more affected than men by all infections except syphilis. Younger soldiers, aged ≤ 24 years, are affected by most of the diseases at higher rates than of any other age group.^12^

Women are a growing number within the Armed Forces; however, militarism is still a male-dominated space in which the woman has a marked place as the wife and partner of the military husband.^13–16^

In the Brazilian Army, female soldiers make up 3.2% of the Force’s personnel and possess 5.35% of the assets in the military garrison of Campinas. Compared to the British Armed Forces, in which 10% of the personnel are women, Brazil is still far behind the female contingent of other nations.^17^

## OBJECTIVE

To assess the prevalence of STIs and the socioepidemiological profile of women in the military garrison of Campinas.

## METHODS

This was a prospective, cross-sectional epidemiological study involving women who attended the Medical Center of the Military Garrison of Campinas from 2017 to 2020 by free and spontaneous demand. The study was approved by the Universidade Federal de São Paulo Ethics Committee (number 2.580.983) on April 4, 2018.

Initially, 1,019 women who consulted at the Medical Center of the Military Garrison of Campinas were selected. They were military personnel or wives and/or companions of military users of the Brazilian Army Health Fund, who had an active or previously active sexual life, of which 647 were allocated to the study. Civil servants, women with no past or present sexual life, and those who had acquired any of the infections, as evaluated through non-sexual means, were excluded. For comparison purposes, two groups were established: 1) the GENERAL Group, which corresponded to the 647 women allocated to the study, and 2) the WITH-STI group, which corresponded only to those who were diagnosed with STIs during the study.

The participants underwent directed anamnesis, complete physical examination, cervico-vaginal material collection, and blood collection for serological tests.

Due to the financial and logistical characteristics of the gynecology service in the military garrison of Campinas, the collection of material for the examination of cervico-vaginal oncotic colpocytology was performed in a conventional environment. For statistical analysis of the cytological findings, three results were established: negative, minor cytological alterations that corresponded to the results of squamous cell atypia of undetermined significance (ASCUS), and low-grade intraepithelial lesion (LIEBG); cytological alterations greater than these corresponding to the results of squamous cell atypia that did not exclude high-grade (ASC-H), glandular cell atypia (AGC), and high-grade intraepithelial lesion (LIEAG). There were no cases of cancer; therefore, we did not compute this finding.

Colposcopy was performed only in women with altered cytology results or visual alterations of the cervix on gynecological examination. The terminology used followed the norms of the International Federation of Cervical Pathology and Colposcopy.^18^ In the presence of abnormal findings, a biopsy was performed with punch-hole forceps, and the collected material was deposited in a vial containing a formaldehyde solution and sent for pathological study.

All the molecular tests were automated. To perform human papillomavirus (HPV) hybrid capture and real-time polymerase chain reaction tests for the diagnosis of *Chlamydia trachomatis* and *Neisseria gonorrhoeae*, the liquid seeding medium Cellpreserv from KOLPLAST (Itupeva, Brazil) was used. Two samples were collected from each woman and placed in separate Cellpreserv vials, because the analysis methodology for HPV was different from that for *Chlamydia trachomatis*/*Neisseria gonorrhoeae*.

Serological blood samples were collected by peripheral vein puncture in the internal laboratory at the Medical Center of the Military Garrison of Campinas and sent to the central laboratory of the São Paulo Military Hospital for automated analysis.

In the statistical analysis, frequency comparisons were performed using the chi-square test or Fisher’s exact test, where necessary. The significance level was set to 0.05, and statistical analysis was performed using Minitab-19 software. Among the analyses and data crossing between the variables, the cutoff age of 25 years was chosen in view of the recommendations for *Chlamydia trachomatis* and *Neisseria gonorrhoeae* screening in women aged 14–25 years and ˃ 25 years for cervical cancer.

## RESULTS

The 647 women included in this study were military personnel, sexual partners of military personnel, and dependents. The mean age was 38 years ± standard deviation (SD) 16.13. Their minimum and maximum ages were 14 and 93 years, respectively. The analysis was conducted from 2017 to 2020. In the last year, there was a significant decrease in visits, with a consequent decrease in the number of women who could be evaluated due to the beginning of the coronavirus disease-2019 pandemic.

In the general population studied, the prevalence of STIs, in increasing order of incidence, was 0% for HIV, 0.3% for hepatitis B and C (2 cases), 0.62% for syphilis (4 cases), 0.62% for gonorrhea (4 cases), 1.08% for chlamydia (7 cases), and 2.63% for HPV (17 cases). As there were no cases of HIV infection, this information has been removed from the tables and graphs. The frequency of infection found in the group of 30 women with 34 diagnosed STIs was 5.89% for hepatitis B and C, 11.77% for syphilis, 11.77% for gonorrhea, 20.58% for chlamydia, and 50% for HPV (Table 1).

**Table 1:** Sexually transmitted infection prevalence in the general population

	Disease	Prevalence n = 647 (%)	Frequency n = 34 (%)	Number of cases n = 647
**General population** **(n = 647)**	**HBsAg**	0.15	2.94	1
	**HCV**	0.15	2.94	1
	**Syphilis (VDRL)**	0.62	11.77	4
	**Hybrid Capture HPV**	2.63	50	17
	**Chlamydia (PCR-RT)**	1.08	20.58	7
	**Gonorrhea (PCR-RT)**	0.62	11.77	4

HBsAg = hepatitis B antigen; HCV = hepatitis C virus; VDRL = Venereal Disease Research Laboratory; HPV = human papillomavirus; PCR-RT = reverse transcription followed by polymerase chain reaction.

In the general epidemiological assessment, the largest number corresponded to the dependents of the military, with 479 women (74.03%), followed by 117 (18.08%) full-time military women, and finally, 51 (7.88%) pensioners.

The group aged 25 years or older had the highest absolute number of women (n = 480) and the most frequent relationship presented in this same group was “dependents” of military personnel (73.95%), with a statistically significant result (P = 0.00001).

About rank or graduation, in the lowest age group, there was a preponderance of “soldiers” (63.47%) and in the highest, there was an almost equal division between “enlisted” and “officers,” with 52.7% and 47.29%, respectively (P = 0.0159). Thus, according to the hierarchical circle to which the woman belonged, we had a general preponderance of the “enlisted” circle, with about 11% more than the circle of officers. Regarding age groups, the largest proportion of the enlisted circle was more evident in the group of people under 25 years old, with almost 30% more than the circle of officers. In the older age group, the proportions were similar between the two groups. The results were statistically significant for these data (Table 2).

**Table 2:** Distribution of women in groups, according to the post and link to the Brazilian Army, according to age group

	Variable		< 25 (n = 167)		³ 25 (n = 480)		P*
**General group**	**Hierarchical circle**						
		Officers	61	(36.52%)	227	(47.29%)	0.0159*
		Squares	106	(63.47%)	253	(52.7%)	
	**Link with Army**						
		Dependent on military	124	(72.25%)	355	(73.95%)	< 0.0001*
		Holder	41	(24.55%)	76	(15.83%)	
		Pensioner	2	(1.19%)	49	(10.2%)	
	**Variable**		**< 25 (n = 18)**		**³ 25 (n = 12)**		**P** *
**Group with sexually transmitted infections**	**Hierarchical circle**						
		Officers	5	(27.78%)	3	(25%)	0.8661
		Squares	13	(72.22%)	9	(75%)	
	**Link with Army**						
		Dependent on military	12	(66.67%)	11	(91.67%)	
		Holder	6	(33.33%)	1	(8.33%)	0.2751
		Pensioner	0	(0%)	0	(0%)	

* Pearson’s Chi-square test.

Of the 30 women diagnosed with STIs, 18 were younger than 25 years and 12 were 25 years or older. The largest number also corresponded to military dependents, with 23 women (76.67%), followed by seven (23.33%) full-time military personnel and, finally, no pensioners, but without statistical significance between the types of attachment, hierarchical circles, and age groups (Table 2). The mean age in this group was 36.9 years (± SD 10.46), with a minimum and a maximum age of 17 and 59 years, respectively.

By subtracting the infected women from the 647 women studied, we created a WITHOUT-STI group with 617 women. We then compared the WITHOUT-STI group (n = 617) with the WITH-STI group (n = 30).

In the WITH-STI group, more than half of the population studied was nulliparous, the rest had between one and three children, and no woman had more than three children. The number of abortions was almost five times higher in the WITHOUT-STI group, which was a statistically significant finding (P = 0.00007) (Table 3).

**Table 3:** Distribution of the epidemiological profile of the 647 women treated at the Medical Post of the Military Garrison of Campinas gynecology outpatient clinic, according to the presence of Sexually Transmitted Infections

Variable		Without STI (n = 617)		With STI (n = 30)		P*
**Parity**						
	0	239	(38.73%)	18	(60%)	0.08601
	1–3	325	(52.67%)	12	(40%)	
	4–7	53	(8.59%)	0	(0%)	
**Number of abortions**						
	0	327	(53%)	27	(90%)	0.00007*
	≥ 1	290	(47%)	3	(10%)	
**Number of partners**						
	1–5	443	(71.80%)	21	(70%)	0.00343*
	6–10	37	(6%)	3	(10%)	
	≥ 11	9	(1.46%)	3	(10%)	
	Unknown/unanswered	128	(20.74%)	3	(10%)	
**Marital status**						
	Married / Stable union	374	(60.62%)	14	(46.67%)	0.12781
	Single / Widow	243	(39.38%)	16	(53.33%)	
**Sexual onset**						
	≤ 14	43	(6.97%)	4	(13.33%)	0.20125
	15–17	198	(32.09%)	13	(43.33%)	
	≥ 18	342	(55.43%)	13	(43.33%)	

* Pearson’s Chi-square test; STI = sexually transmitted infection.

The number of partners was restricted to 10 for approximately 80% of women in both groups. However, the percentage of women in the WITH-STI group was approximately seven times higher (P = 0.00343) when they had 11 or more partners (Table 3).

Most women in the WITHOUT-STI group had established relationships, while most women in the WITH-STI group did not have a steady partnership, though this data was not statistically significant (Table 3).

An earlier age of sexual initiation (17 years or less) favored the presence of STIs about 1.5 times more and almost 2 times more when sexarche occurred at 14 years or less. (Table 3)

Most women (75%) were asymptomatic and went to consult for routine exams. Oncotic colpocytology was performed in 483 women, who were 25 years of age or older and others who showed a desire to undergo the examination. When cervico-vaginal cytological evaluation was performed, the results showed 410 (84.89%) normal examinations, 68 (14.08%) with minor alterations, and 5 (1.03%) with major alterations. There were only cases of ASCUS, LIEBG, and LIEAG, and no cases of AGC or ASC-H. Of the 73 colposcopies resulting from altered cytology, 60 (82.2%) were normal, 12 (16.44%) had minor findings, and 1 (1.37%) had major findings. Of the 13 biopsies generated, 7 (53.84%) had a report of chronic cervicitis, 5 (38.46%) had low-grade lesions, and 1 (7.69%) had a high-grade lesion. Thus, after colpohistological confirmation, approximately 1% of the 483 women were diagnosed with a low-grade lesion in the uterine cervix and 0.21% with a high-grade lesion as the final diagnosis.

There were three cases of concomitant STIs, totaling 34 infections in 30 women: one woman with HPV associated with Chlamydia and Gonococcus and, the other two with Chlamydia and Gonococcus.

**Graph 1:**
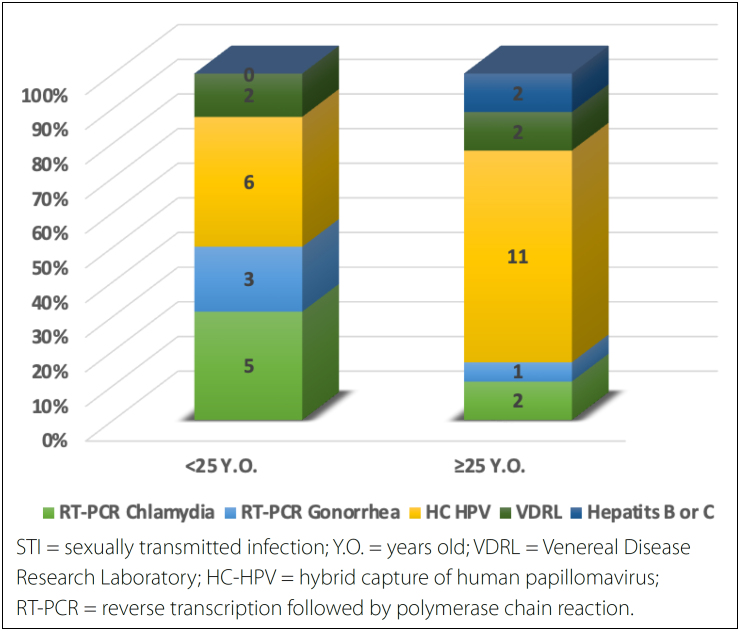
Numeric comparison among women with STIs in relation to age group.

In the GENERAL population, the ratio of Chlamydia to gonorrhea infection in both age groups tended toward 2:1. The group of patients younger than 25 years showed a tendency toward a higher proportion of Chlamydia and Neisseria, as well as syphilis and HPV, and a lower proportion for hepatitis. Chlamydia was more frequent in women under 25 years of age (P = 0.0144), while gonorrhea showed a similar trend (P = 0.0549). (Table 4, Graph 1)

**Table 4:** General distribution of test results according to age group in the general population (n = total of 647) and with sexually transmitted infections (n = 30)

		Variable		< 25 (n = 167)		≥ 25 (n = 480)		P*
**General (n = 647)**	**HBsAg**						
		Positive	0	(0%)	1	(0.21%)	
		Negative	167	(100%)	479	(99.79%)	1.0000
	**HCV**						
		Positive	0	(0%)	1	(0.21%)	1.0000
		Negative	167	(100%)	479	(99.79%)	
	**Syphilis (VDRL)**						
		Positive	2	(1.20%)	2	(0.42%)	0.2752
		Negative	165	(98.8%)	478	(99.58%)	
	**Hybrid capture HPV**						
		Positive	6	(3.59%)	11	(2.29%)	0.4005
		Negative	161	(96.4%)	469	(97.71%)	
	**Chlamydia (PCR-RT)**						
		Positive	5	(2.99%)	2	(0.42%)	0.0144*
		Negative	162	(97%)	478	(99.58%)	
	**Gonorrhea (PCR-RT)**						
		Positive	3	(1.79%)	1	(0.21%)	0.0549
		Negative	164	(98.2%)	479	(99.79%)	
	**Variable**		**< 25 (n = 18)**		**³ 25 (n = 12)**		**P** *
**With sexually transmitted** **infections** **(n = 30)**	**HBsAg**						
		Positive	0	(0%)	1	(8.33%)	
		Negative	18	(100%)	11	(91.67%)	
	**HCV**						0.76508
		Positive	0	(0%)	1	(8.33%)	
		Negative	18	(100%)	11	(91.67%)	
	**Syphilis (VDRL)**						
		Positive	2	(11.11%)	2	(16.67%)	0.66100
		Negative	16	(88.89%)	10	(83.33%)	
	**Hybrid capture HPV**						
		Positive	6	(33.33%)	11	(91.67%)	0.00158*
		Negative	12	(66.67%)	1	(8.33%)	
	**Chlamydia (PCR-RT)**						
		Positive	5	(27.78%)	2	(16.67%)	0.48086
		Negative	13	(72.22%)	10	(83.33%)	
	**Gonorrhea (PCR-RT)**						
		Positive	3	(16.67%)	1	(8.33%)	0.51067
		Negative	15	(83.33%)	11	(91.67%)	

* Pearson’s Chi-square test.

HBsAg = hepatitis B antigen; HCV = hepatitis C virus; VDRL = Venereal Disease Research Laboratory; HPV = human papillomavirus; PCR-RT = reverse transcription followed by polymerase chain reaction.

When the same age comparison in women in the WITH-STI group was made, the group of younger women under 25 years of age also tended to have a higher proportion of Chlamydia and Neisseria but lower proportions of syphilis and hepatitis. HPV was significantly more prevalent in older women than in younger women (P = 0.00158).

## DISCUSSION

This study is unprecedented in the Brazilian Armed Forces. Previous studies have only involved young, conscripted men entering mandatory military service.

The military garrison of Campinas consists of more than 7,000 beneficiaries of the Brazilian Army Health Fund, including approximately 2,600 active-duty military men and women.

Most of the women treated as outpatients sought preventive gynecological consultations, and 75% of them were asymptomatic. These data are consistent with the profile of the Brazilian female population, which is more concerned about health than the male population. Genital discharge was the most frequent complaint, particularly in younger patients.

Due to the type of activity developed, some military women and companions of military men are more likely to be involved in high-risk sexual practices and contract STIs for the following reasons: less use of barrier contraception, little knowledge about gynecological health, and lower education level.^18–21^

However, a bias that we can point out for the population studied is the fact that the military garrison is located in a region of greater purchasing and educational power with a high human development index (the Campinas region), which may explain the low number of diagnosed STIs, regardless of age. It is also a population that has adequate guidance on preventive measures, in addition to being a peace army.

In this study, majority of the women (almost 56%) made up the hierarchical circle of the squares. This is because to be recruited in this class, only a technical high school or elementary educational level is required. For the rank of officers, a higher educational level is required.

The highest prevalence of stable relationships was directly proportional to the absence of STIs, with 60.62% in the WITHOUT-STI group. However, in the WITH-STI group, there was an inversion of this proportion, with 53.33% of women without a steady partnership, thus consistent with the greater presence of STIs in the groups where there was greater turnover in the partnership.

In the evaluation of the 647 women, most of them (54.87%) started sexual activity later, when they were 18 years old or older. This is probably because they have more stable habits infringed upon by military customs, which may have been a protective factor against STIs.

Early sexual intercourse favored the presence of STIs, given that 56.67% of women with STI had sexual intercourse before 17 years of age. Thus, corroborating what is known about early sexual initiation and the inconsistent use of condoms during the first sexual intercourse could leave adolescents in a situation of greater vulnerability.

In this line, if we consider women up to 17 years of age as adolescents, we have approximately 40% of all women starting their sexual activity in adolescence and less than 7% of girls starting their sexual activity as early as 14 years of age. This number is lower than the 10% reported in a recent study carried out in Italy.^22^

A more constant behavior was observed with regard to the partnership, since more than 70% of the women had between one and five partners. In general, this picture reflects an expected “family model,” operated by the Army, which reflects the case of families in which only the husband is in the military. This is different from the US military, where nearly 60% of women report having more than one sexual partner per year. A separate study revealed that 27% of female service members interviewed had more than one partner in the previous 90 days. In this study, only 17% reported regular condom use. It is noteworthy that there is an important difference in the behavior of the military in periods of war compared to periods of peace, since war is still considered a harbinger of STIs today.^23–26^

According to the World Health Organization, adolescence is a fundamentally biological process that occurs in individuals aged between 10 and 19 years. A survey found that 49.25% of the investigated adolescents had already started their sexual lives. The occurrence of the first intercourse before 15 years of age was observed in approximately 30% of these individuals. These data are similar to those of other studies that revealed that most adolescents experience their first sexual intercourse at this age. Early sexual initiation is considered a risk factor, as is the number of sexual partners exposed to STIs.^23^

Like the GENERAL population, the STI group in its entirety (100%) had between 0 and 3 children, 60% of whom were nulliparous. Therefore, the presence of STIs did not increase the number of abortions, since women without STIs had approximately five times more abortions than women with STIs. Again, military habits that involve military families seem to favor family planning as well as the prevention of STIs. Additionally, of the 483 women, approximately 1% were diagnosed with a low-grade lesion in the uterine cervix and 0.21% with a high-grade lesion as the final diagnosis.

There were three cases of concomitant STIs, totaling 34 infections in 30 women: one woman with HPV associated with Chlamydia and Gonococcus and the other two with Chlamydia and Gonococcus.

The ratio of Chlamydia to gonorrhea infection in both age groups tended toward 2:1. The group of patients younger than 25 years showed a tendency toward a higher proportion of every sexual disease studied except for hepatitis. Chlamydia was more frequent in women under 25 years of age (P = 0.0144), while gonorrhea showed a similar trend (P = 0.0549). (Table 4, Graph 1)

When the same age comparison in women with STIs was made, HPV was significantly more prevalent in older women than in younger women (P = 0.00158).

## CONCLUSION

The present study with women who consulted at the Medical Center of the Military Garrison of Campinas between 2017 and 2020 showed a low prevalence of STIs, with 4.64% of the studied population infected by at least one agent surveyed. The combined prevalence rates were 0.30% for hepatitis B and C, 0.62% for syphilis, 1.08% and 0.62% for Chlamydia and Gonococcus, respectively, and 2.62% for HPV. There were no cases of HIV and no statistically significant differences in the prevalence rates of Hepatitis B and C, syphilis, or HPV infections. Regarding Chlamydia infection, there was a statistically significant difference in the prevalence in women under 25 years of age, and Neisseria infection followed the same trend. Early sexual intercourse prevailed in the younger age group and favored the presence of STIs in both age groups.
